# Hsa-miR-329 exerts tumor suppressor function through down-regulation of *MET* in non-small cell lung cancer

**DOI:** 10.18632/oncotarget.7517

**Published:** 2016-02-19

**Authors:** Cheng-Cao Sun, Shu-Jun Li, Feng Zhang, Jing-Yu Pan, Liang Wang, Cui-Li Yang, Yong-Yong Xi, De-Jia Li

**Affiliations:** ^1^ Department of Occupational and Environmental Health, School of Public Health, Wuhan University, Wuhan, P. R. China; ^2^ Wuhan Hospital for the Prevention and Treatment of Occupational Diseases, Wuhan, P. R. China

**Keywords:** hsa-miRNA-329(miR-329), MET, non-small cell lung cancer (NSCLC), proliferation, apoptosis

## Abstract

MicroRNAs (miRNAs) act as key regulators of multiple cancers. Hsa-miR-329 (miR-329) functions as a tumor suppressor in some malignancies. However, its role on lung cancer remains poorly understood. In this study, we investigated the role of miR-329 on the development of lung cancer. The results indicated that miR-329 was decreased in primary lung cancer tissues compared with matched adjacent normal lung tissues and very low levels were found in a non-small cell lung cancer (NSCLC) cell lines. Ectopic expression of miR-329 in lung cancer cell lines substantially repressed cell growth as evidenced by cell viability assay, colony formation assay and BrdU staining, through inhibiting cyclin D1, cyclin D2 and up-regulatiing p57(Kip2) and p21(WAF1/CIP1). In addition, miR-329 promoted NSCLC cell apoptosis, as indicated by up-regulation of key apoptosis gene cleaved caspase-3, and down-regulation of anti-apoptosis gene Bcl2. Moreover, miR-329 inhibited cellular migration and invasiveness through inhibiting matrix metalloproteinases (MMP)-7 and MMP-9. Further, oncogene *MET* was revealed to be a putative target of miR-329, which was inversely correlated with miR-329 expression. Furthermore, down-regulation of MET by siRNA performed similar effects to over-expression of miR-329. Collectively, our results demonstrated that miR-329 played a pivotal role in lung cancer through inhibiting cell proliferation, migration, invasion, and promoting apoptosis by targeting oncogenic *MET*.

## INTRODUCTION

Lung cancer is the leading cause of cancer-related deaths both in men and women around the world for several decades. There are estimated to be 1.8 million new cases in 2012 (12.9% of the total), killing about 1.59 million (19.4% of the total) people per year globally, extrapolating from a 2012 International Agency for Research on Cancer (IARC) risk assessment [1]. Approximately 80% of lung cancers are classified histopathologically as non-small cell lung carcinomas (NSCLC). At early stages of NSCLC, the only treatment is surgery, with a 5-year overall survival rate of 40% [2], whereas chemotherapy is mostly employed for small cell lung cancer (SCLC). These changes are attributed to silencing of tumor suppressor genes, dysregulation of proto-oncogenes, and an up-regulation of genes that promote cell growth and transformation and ultimately tumor development [3].

In recent years, there has been a considerable interest in understanding the role of microRNAs (miRNAs) on tumor development. MiRNAs, a class of ~20 - 23 nucleotide (nt) noncoding RNAs, repress the expression of their target genes through binding mRNAs at specific sequences. They control gene expression at post-translational level by various mechanisms, such as decay of mRNAs and blockage of translation [4-6]. MiRNAs binding to the 3′-untranslated region (3′-UTR) of target mRNAs leads to translational repression or degradation of mRNA. MiRNAs might serve as new therapeutic strategies for cancers, as they can act as tumor suppressors or as oncogenes dependent on their target mRNAs [7-9]. In primary lung cancer, miRNA expression profiles have been studied, and several miRNA clusters have been investigated [10-12]. MiRNAs are thought to play critical roles on cell proliferation, migration and invasion in non-small cell lung cancer [13-16]. MiR-329 is located on 14q32.31 and is down-regulated in several cancers, including neuroblastoma [17-19], gastric cancer [20] and so on. Moreover, Xiao *et al*. reported that miR-329 was down-regulated in glioma, and inhibited cell proliferation of glioma cells by regulating E2F1-mediated inhibition of Akt pathway [21]. These results reveal the powerfully inhibitory role of miR-329 on tumorigenesis. However, the function and molecular mechanism of miR-329 in human NSCLC are still elusive.

In this study, we show for the first time that miR-329 directly targets and regulates the full-length 3′-UTR of the human MET mRNA, which is up-regulated in many cancers, including lung cancer. c-Met is encoded by *MET* gene, and plays a key role on the control of invasive growth not only during tumorigenesis but also in embryonic development, organ development, and inflammatory response [22]. Here, we reported that miR-329 was indeed suppressed in primary lung cancers tissues compared with the matching normal lung tissues, and found 3′-UTR of the human MET mRNA is really a target of miR-329. Collectively, we discovered that miR-329 exerted its tumor suppressive effects on non-small cell lung cancer *in vitro* and *in vivo* by directly targeting the 3′-UTR of MET mRNA.

## RESULTS

### MiR-329 is down-regulated in primary human lung cancer

**Figure 1:**
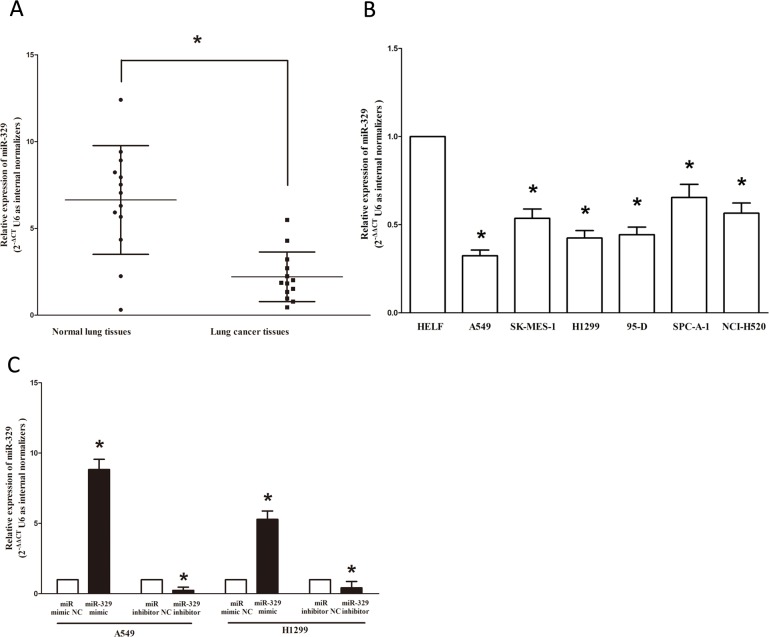
Expression miR-329 is significantly down-regulated in primary human lung cancer and NSCLC cell lines **A.** miR-329 is significantly decreased in primary human lung cancer tissues in comparison to matched-normal lung cancer tissues. *n* = 13 for each group. **B.** The expression level of miR-329 in five NSCLC cell lines and normal HELF cells. Assays were performed in triplicate. **C.** The expression of miR-329 in A549 and H1299 cells after transfection for forty-eight hours. Means ± SEM are shown. Statistical analysis was conducted using student *t*-test.

To determine whether miR-329 is down-regulated in lung cancer, we measured the mature miR-329 level in human primary lung tumors (NSCLC) and pair-matched lung tissues by qRT-PCR. We used U6 that is not deregulated in lung cancer for normalization. The results showed that miR-329 expression in the tumors was significantly (*p* < 0.001) reduced in 13 lung cancers relative to their matched controls among 13 samples analyzed (Figure 1A). Next, we examined miR-329 expression in NSCLC cell lines, and results demonstrated a lower expression of miR-329 in A549, SK-MES-1, SPC-A-1, H1299, 95-D and NCI-H520 cell lines, compared with that of in normal lung cells HELF (Figure 1B and Figure S1A). Among the six NSCLC cell lines, miR-329 decreased the most in A549 and H1299 cell lines, thus, we chose A549 and H1299 for model of NSCLC cell lines. Moreover, to evaluate the clinical significance of miR-329, we assessed the associationof its expression with clinic-pathological parameters (i.e., stage, maximum diameter and lymph node metastasis). Results demonstrated miR-329 expression levels in NSCLC were significantly associated with tumor size (*P* = 0.0079), TNM stage (*P* = 0.0048) and lymph node metastasis (*P* = 0.0162). However, miR-329 expression was not associated with other clinical characteristics such as differentiation (*P* = 0.7558), gender (*P* = 0.1696), smoking history (*P* = 0.2164), age (*P* = 0.0895) or histological tumor type (*P* = 0.9512) in NSCLC (Table 1). In addition, we transfected A549 and H1299 cells with miR-329 mimic or miR mimic NC, and miR-329 inhibitor or miR-329 inhibitor NC, separately. Results indicated that miR-329 mimic significantly promoted the expression of miR-329, and miR-329 inhibitor suppressed the expression of miR-329 (Figure 1C). Thus, it was concluded that the decreased expression of miR-329 might play an important role in lung cancer progression and development.

**Table 1:** Correlation between miR-329 expression and clinicopathological parameters of NSCLC patients(n=26)

Parameter	n	Relative miR-329 expression		
		Low	High	*P*-value
Age/years				0.0895[b]
≤ 65	8	2	6	
> 65	18	12	6	
Gender				0.1696[b]
Male	20	9	11	
Female	6	5	1	
Differentiation				0.7558[a]
Well, moderate	16	9	7	
Poor	10	5	5	
Tumor size (maximum diametercm)				0.0079[*][b]
≤ 3cm	14	4	10	
> 3cm	12	10	2	
Smoking history				0.2164[b]
Smokers	18	8	10	
Never smokers	8	6	2	
Lymph node metastasis				0.0162[*][b]
Positive	15	11	3	
Negative	11	3	9	
TMN stage				0.0048[*][b]
I	13	3	10	
II/III/IV	13	11	2	
Histological tumor type				0.9512[a]
Squamous cell carcinoma	11	6	5	
Adenocarcinoma	15	8	7	

a Chi-square test

b Fisher's exact test

* P < 0.05

### Expression of c-met is up-regulated in primary human lung cancer

**Figure 2:**
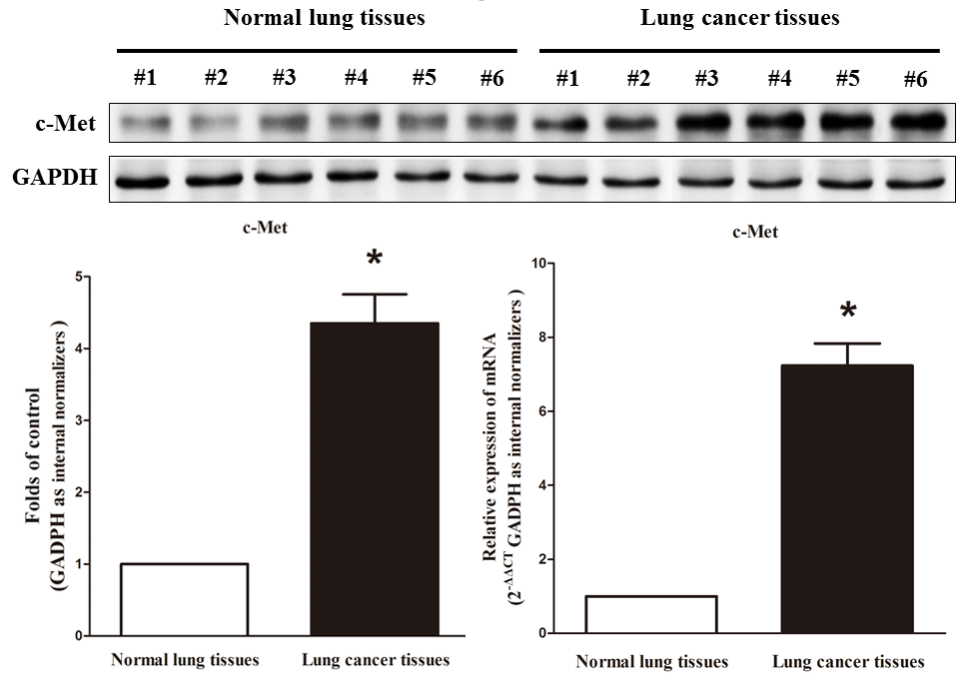
c-Met is up-regulated in primary lung cancer Western-blot of c-Met protein and qRT-PCR of c-Met mRNA in lung cancer tissues and normal lung cancers. *n* = 13 for each group. Means ± SEM are shown. Statistical analysis was conducted using student *t*-test.

c-Met is important oncogene that shown strong power of oncogenicity, by promotion of cell growth, migration, invasion and epithelial mesenchymal transition (EMT), as well as inhibition of cell apoptosis in many tumors including lung cancer. Thus, we next examined c-Met expression in human primary lung tumors (NSCLC) and pair-matched lung tissues, and our western blot results demonstrated that the expression of c-Met protein is increased in lung cancer tissues compared with normal lung tissues (Figure 2). These results were confirmed by qRT-PCR of c-Met mRNA expression (Figure 2). Since c-Met is the target protein of an important proto-oncogene MET, aberrations of its protein might contribute to human lung cancer.

### Silence of *MET* expression inhibits lung cancer cell growth, migration, invasion and apoptosis

**Figure 3:**
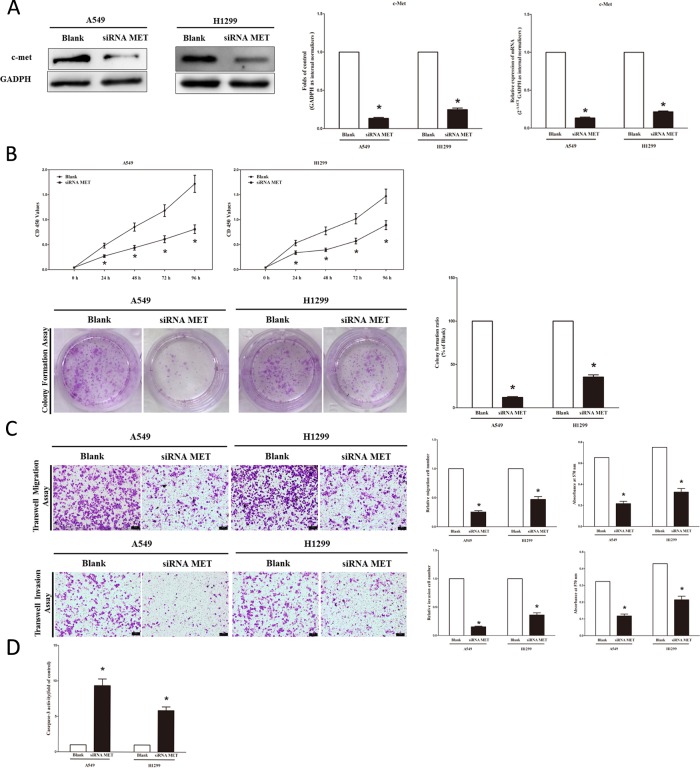
Silence of *MET* expression inhibits lung cancer cell growth, migration, invasion and apoptosis **A.** Western-blot of c-Met protein in siRNA *MET* treated and blank A549 and H1299 cells. **B.** CCK8 assays of A549 and H1299 cells after transfected (un-transfected) with siRNA *MET*; Shown are representative photomicrographs of colony formation assay after transfected with (without) siRNA *MET* for ten days. **C.** Shown are representative photomicrographs of tanswell migration assay after transfected with (without) siRNA *MET*; Shown are representative photomicrographs of tanswell invasion assay after transfected with (without) siRNA *MET*. **D.** Quantitative representation of casepase-3 activity in A549 and H1299 cells transfected with (without) siRNA *MET* for forty eight hours. Assays were performed in triplicate. Means ± SEM are shown. Statistical analysis was conducted using student *t*-test.

We next examined the potential tumorigenicity of *MET* in lung cancer. Silence of *MET* expression by siRNA *MET* significantly inhibited the expression of c-Met (Figure 3A). Moreover, loss of *MET* expression also contributed to inhibition of lung cancer cell (both A549 and H1299 cells) growth (Figure 3B), migration and invasion (Figure 3C), and promotion of cell apoptosis in lung cancer cell (both A549 and H1299 cells) (Figure 3C). These results further verified the powerful tumorigenicity of *MET* in lung cancer. Thus, we adopted *MET* for as targeted oncogenes.

### MiR-329 targets human *MET*

**Figure 4:**
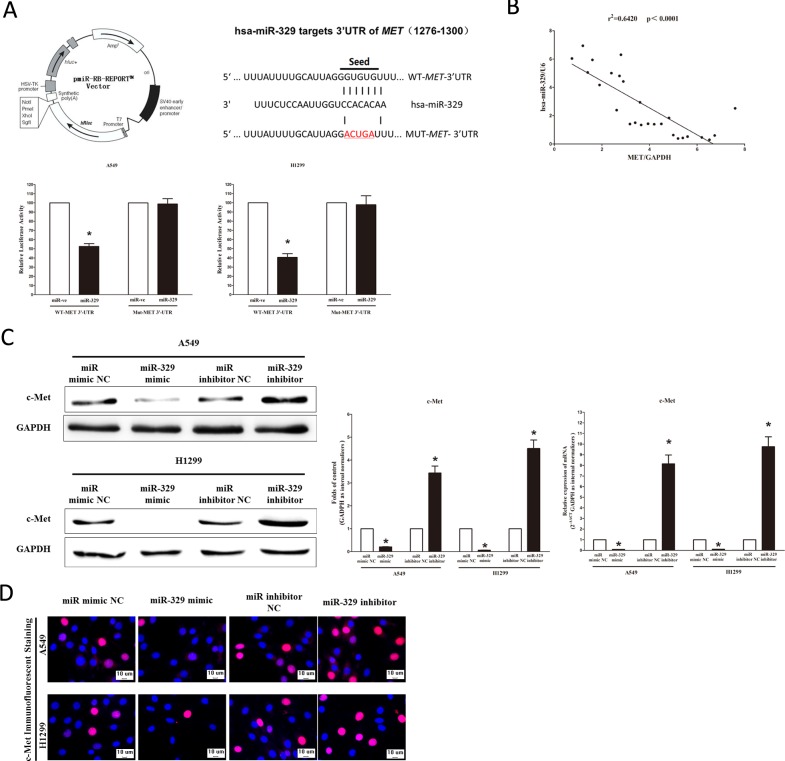
*MET* proto-oncogene is a target of miR-329 at specific 3′-UTR site **A.** pmiR-RB-REPORT TM dual-luciferase reporter vector; The 3′-UTR of *MET* harbors one miR-329 cognate site; Relative luciferase activity of reporter plasmids carrying wild-type or mutant *MET* 3′-UTR in A549 and H1299 cells co-transfected with negative control (NC) or miR-329 mimic. **B.** Scatter plots showing the inverse association between miR-329 level and MET mRNA expression. **C.** Western-blot of c-Met protein in A549 and H1299 cells after transfection; statistical analysis of western-blot; qRT-PCR of c-Met mRNA in A549 and H1299 cells after transfection. **D.** c-Met immunofluorescent staining in A549 and H1299 cells after transfection. Bar = 10 μm. Assays were performed in triplicate. Means ± SEM are shown. Statistical analysis was conducted using student *t*-test.

We then explored the underlying molecular mechanism of the antitumorigenic property of miR-329 in lung cancer cells. Since miRNAs primarily mediate their biological functions in animal cells by impeding the expression of target genes, we searched different data bases (TargetScan,microRNA.org and PicTar) for its potential targets that exhibited oncogenic properties. MET (hepatocyte growth factor receptor), which harbors one conserved miR-329 cognate sites, namely, 1276-1300 of *MET* 3′-UTR) (Figure 4A), is a predicted target of miR-329. To determine whether *MET* expression are indeed regulated by miR-329, the *MET* 3′-UTR were cloned into a luciferase reporter plasmid (Figure 4A), and the ability of miR-329 to inhibit expression of the adjacent hRluc coding region was quantified. For this purpose, the luciferase reporter plasmid pmiR-RB-REPORT ^TM^-*MET*-3′-UTR or a mutant reporter plasmids carrying point mutations in the putative miR-329 binding sites was co-transfected with miR-329 mimics or miR mimic NC, separately. The results show that miR-329 suppresses luciferase activity by approximately 50% in A549 cells and 58% in H1299 cells when the reporter plasmid carried the wild type *MET* 3′-UTR (Figure 4A), but no significant suppression was observed when the reporter plasmid carried a mutant *MET* 3′-UTR (i.e., pmiR-RB-REPORT ^TM^-mut-*MET*-3′ -UTR). Moreover, we evaluated the correlation between MET mRNA and miR-329 expression in 26 primary NSCLC lung tissues. Expression of MET mRNA and miR-329 exhibited a significant inverse correlation as calculated by Pearson correlation (r = −0.8012, *P* < 0.0001) (Figure 4B). This result further supported that miR-329 targeted to *MET*. We next examined the role of miR-329 on the expression of c-Met. Our results of western blot demonstrated that miR-329 inhibited expression of c-Met protein by approximately 70% and 85%, when compared with blank A549 and H1299 cells (Figure 4C), respectively. These results were confirmed by qRT-PCR analysis (Figure 4C). However, as expected, inhibition of miR-329 increased protein and mRNA expression in A549 and H1299 cells, relative to blank A549 and H1299 cells (Figure 4C), respectively. In addition, our immunofluorescence of c-Met also demonstrated that miR-329 reduced the expression of c-Met in A549 and H1299 cells, and inhibition of miR-329 significantly promoted c-Met expression (Figure 4D). These results suggest that miR-329 binds directly to the predicted binding site in the *MET* 3′-UTR and negatively regulates *MET* expression.

### MiR-329 suppresses tumor growth *in vivo*

**Figure 5:**
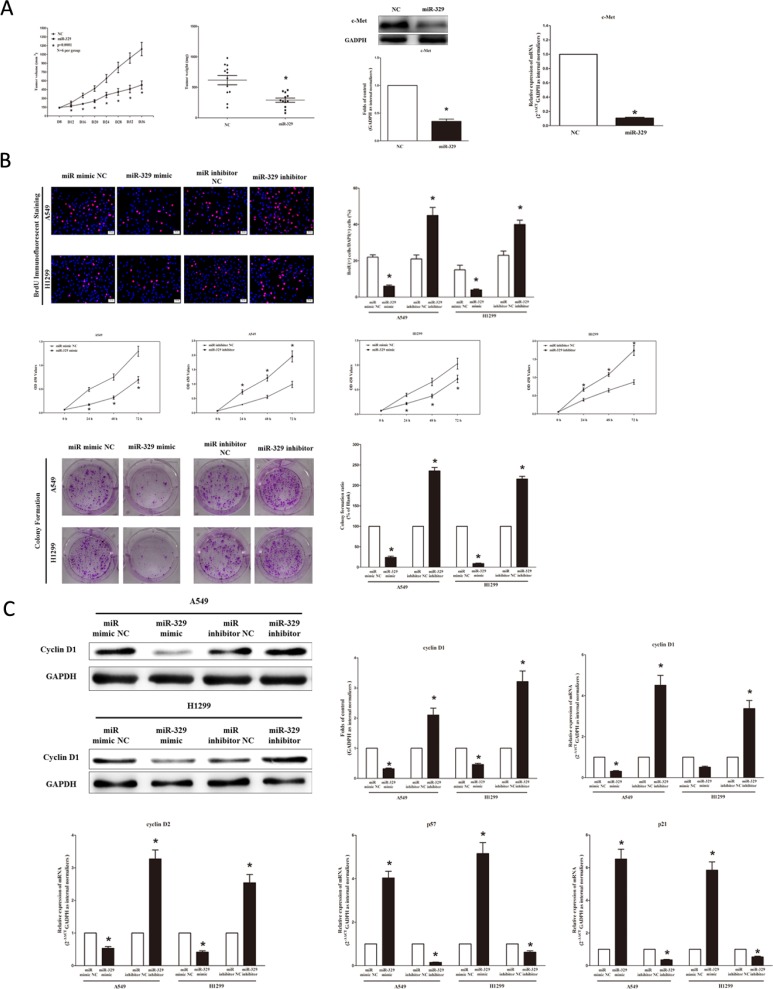
Ectopic expression of miR-329 suppresses tumor growth *in vivo* and *vitro* **A.** Force-expression of miR-329 suppresses tumor growth *in vivo*. Tumor volume and weight in nude mice. Each group contained six mice (*n* = 6); the data are presented as the mean ± SEM; *p < 0.001, compared with the NC group; The expression of c-Met protein and mRNA in nude mice. Assays were performed in triplicate. Means ± SEM are shown. Statistical analysis was conducted using student *t*-test. **B.** Ectopic expression of miR-329 inhibits NSCLC cell growth. A549 and H1299 cells were transfected with miR-329 mimic and mimic NC, miR-329 inhibitor and inhibitor NC, for forty-eight hours, respectively. Shown are representative photomicrographs of BrdU staining in A549 and H1299 cells after transfection. Bar = 100 μm; Statistical analysis of BrdU staining; CCK8 assays of A549 and H1299 cells after transfected with miR-329 mimic, miR-329 mimic NC, miR-329 inhibitor, miR-329 inhibitor NC; Shown are representative photomicrographs of colony formation assay after transfected with miR-329 mimic, miR-329 mimic NC, miR-329 inhibitor or miR-329 inhibitor NC for ten days; Statistical analysis of colony formation assay. **C.** Western-blot of cyclin D1 protein in A549 and H1299 cells after transfection; Statistical analysis of western-blot of cyclin D1 protein; qRT-PCR of cyclin D1, cyclin D2, p57 and p21 mRNA in A549 and H1299 cells after transfection. Assays were performed in triplicate. Means ± SEM are shown. Statistical analysis was conducted using student *t*-test.

To confirm the tumor suppressor role of miR-329 *in vivo*, we established a BALB/c nude mouse xenograft model using A549 cells. After 8 days, miR-329 agomir or miR agomir NC was directly injected into the implanted tumor every 4 days for seven times. The tumor volume was measured every 4 days until day 36. The tumor volume and weight of mice treated with miR-329 agomir were significantly reduced relative to that of treated with miR agomir NC (Figure 5A). This result indicates that miR-329 significantly inhibits the tumorigenicity of A549 cells in the nude mouse xenograft model. In addition, our results of western-blot and qRT-PCR demonstrated that the decreased expression of c-Met tumors developed from miR-329-agomir-treated nude mice relative to control tumors (Figure 5A).

### MiR-329 inhibits lung cancer cell proliferation and colony formation

To further investigate the anticancer role of miR-329 in lung cancer, we examined the role of miR-329 on NSCLC cell (A549 and H1299) proliferation. Our results of BrdU staining revealed that miR-329 inhibited A549 and H1299 cell DNA synthesis by approximately 70% (Figure 5B) and 65% (Figure 5B), compared with blank A549 and H1299 cells, respectively. However, miR-329 inhibitor treatment increased A549 and H1299 cell DNA synthesis by approximately 2.0 folds (Figure 5B) and 1.5 folds (Figure 5B) compared with blank A549 and H1299 cells, separately. To verify this result, we also did the CCK8 assay, and results demonstrated that miR-329 over-expression significantly attenuated A549, H1299 and NCI-H520 cells vitality, while loss of miR-329 promoted cell proliferation (Figure 5B and Figure S1B). In addition, we used colony formation assay to investigate the role of miR-329 on clonogenic survival, and results demonstrated miR-329 mimic treatment caused a decrease in the clonogenic survival of A549 cells compared with blank A549 cells (Figure 5B), while miR-329 inhibitor-treated A549 cells showed an significant increase in the clonogenic survival, when compared with blank A549 cells (Figure 5B).

We next examined the influence of miR-329 on the expression of cyclin D1, a well-established human oncogene, which is over-expressed in lung cancer, breast cancer and pancreatic cancer, and over-expression of cyclin D1 is involved in malignant transformation in lung tissue. Our results discovered that miR-329 significantly decreased the protein and mRNA expression of cyclin D1, while loss of miR-139-5p remarkably increased the level of cyclin D1 in A549 and H1299 cells (Figure 5C). cyclin D2 is highly expressed and promotes tumorigenesisin numerous tumors. In our research, the mRNA expression of cyclin D2 was repressed by over-expression of miR-329 (Figure 5C). p57 is a cyclin-dependent kinase inhibitor, and it is considered to be a candidate of tumor suppressor gene that has been implicated in cancers. Our study revealed that the over-expression of miR-329 is a mechanism for the up-regulation of p57 level in NSCLC cell lines (A549 and H1299) (Figure 5C). The cell cycle inhibitor p21 has been shown to inhibit proliferation both *in vitro* and *in vivo*, and introduction of p21 expression constructs into normal and tumor cell lines results in cell cycle arrest in G1. Our study revealed that miR-329 up-regulated p21 level in NSCLC cell lines (A549 and H1299) (Figure 5C).

Collectively, these results clearly revealed that miR-329 markedly inhibited cell growth in lung cancer cells.

### MiR-329 inhibits lung cancer cell migration and invasion

Then, we examined the role of miR-329 on A549 and H1299 cells migration and invasion. Invasion and migration through the basement membrane are characteristics of metastatic cancer cells.

**Figure 6:**
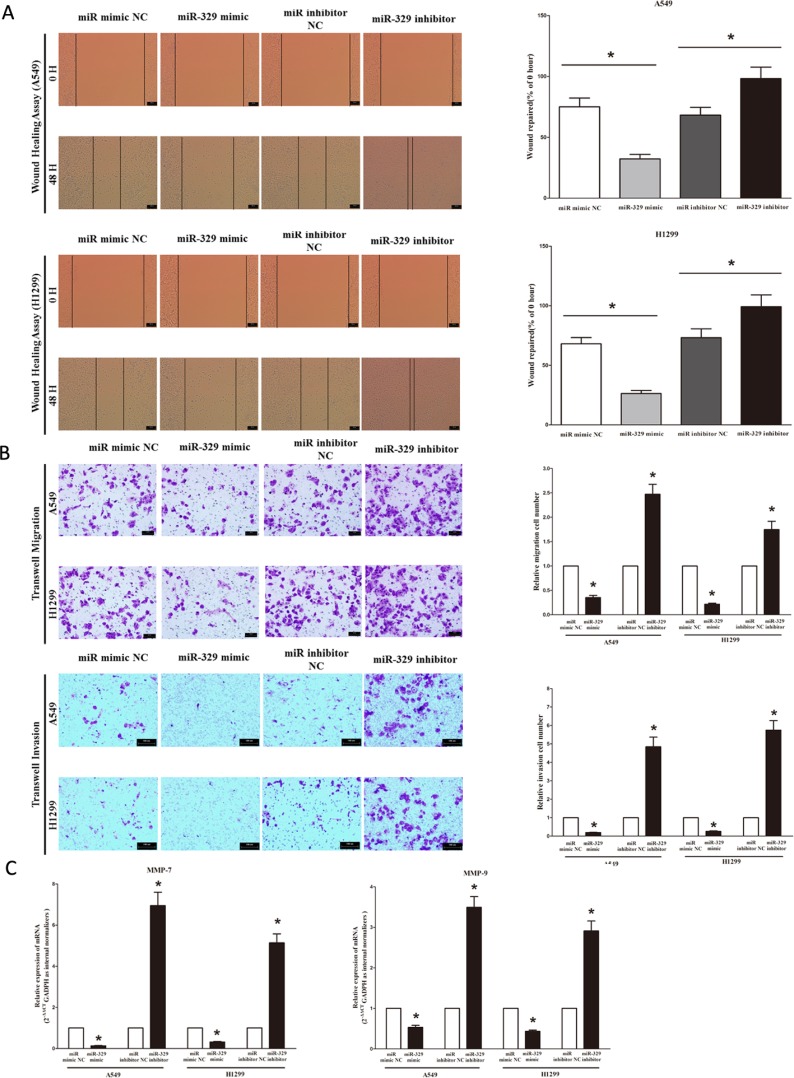
Ectopic expression of miR-329 in A549 and H1299 cells reduces cell migration and invasion motility **A.** Shown are representative photomicrographs of “wound healing assay” in A549 and H1299 cells after transfected miRNAs for 0 hour and forty eight hours. Bar = 100 μm; Statistical analysis of “wound healing assay”. **B.** A549 and H1299 cells were loaded onto the top well of a transwell inserts for cell migration assay. After twenty four hours, cells that migrated to the bottom chamber containing serum-supplemented medium were stained with 0.1% crystal violet, visualized under a phase-contrast microscope, and photographed. Bar = 100 μm; Total number of cells in five fields was counted manually; A549 and H1299 cells were loaded onto the top well of a transwell inserts for cell invasion assay. After twenty four hours, cells that migrated to the bottom chamber containing serum-supplemented medium were stained with 0.1% crystal violet, visualized under a phase-contrast microscope, and photographed. Bar = 100 μm; Total number of cells in five fields was counted manually. **C.** Expression of MMP-7 and MMP-9 mRNA in A549 and H1299 cells after transfection. Assays were performed in triplicate. Means ± SEM are shown. Statistical analysis was conducted using student *t*-test.

We used two different approaches to assess the role of miR-329 on the ability of A549 and H1299 cells migration. In the first technique, we used a “scratch wound healing” assay. Motility of cells at different time points after generation of the wound was monitored under a microscope. Closure of the wound was complete within forty eight hours in control A549 and H1299 cells (Figure 6A). In contrast, miR-329-expressing cells migrated toward the wound at a much slower rate (Figure 6A). In the second approach, cells were seeded in serum-free medium on the top chamber of a two-chamber trans-well cell culture plate, and the cells migrated to the lower chamber containing complete medium after twenty four hours were photographed (Figure 6B) and counted. As expected, migration of miR-329-expressing clones was inhibited by 62% in A549 and 70% in H1299 cells, compared with the blank A549 and H1299 cells (Figure 6B), respectively. However, when treated with miR-329 inhibitor, migration in miR-329-expression defect A549 and H1299 cells were significantly increased by approximately 2.5 and 1.6 folds relative to blank A549 and H1299 cells (Figure 6B), respectively. These results were confirmed in NCI-H520 cell lines (Figure S1C).

To investigate the role of miR-329 on A549 and H1299 cells invasion, we used a transwell invasion assay. As expected, invasion of miR-329-expressing clones was inhibited by 75% in A549 and 56% in H1299 cells, relative to the blank A549 and H1299 cells (Figure 6B), respectively. However, when treated with miR-329 inhibitor, invasion in miR-329-expression defect A549 and H1299 cells were significantly increased by approximately 4.7 and 5.6 folds relative to blank A549 and H1299 cells (Figure 6B), separately. These results were confirmed in NCI-H520 cell lines (Figure S1C).

We also investigated the role of miR-329 on expression of MMP-9 and MMP-7, which all play a key role on tumor metastasis, and results indicated miR-329 inhibited the mRNA expression of MMP-9 and MMP-7 both in A549 and H1299 cells (Figure 6C). As expected, loss of miR-329 significantly increased the mRNA expression of MMP-9 and MMP-7 in both A549 and H1299 cells (Figure 6C).

These results, taken together, clearly demonstrated that miR-329 expression markedly reduces the migration and invasion motility of lung cancer cells.

### MiR-329 promotes lung cancer cell apoptosis

**Figure 7:**
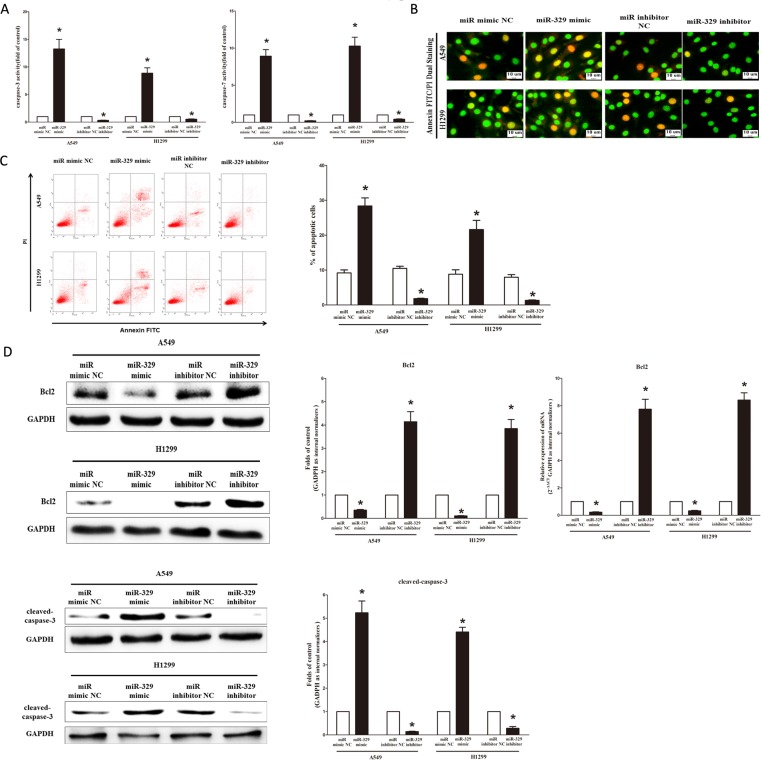
Ectopic expression of miR-329 promotes apoptosis in A549 and H1299 cells **A.** Quantitative representation of casepase-3 and casepase-7 activity in A549 and H1299 cells transfected with related miRNAs for forty eight hours. **B.** Shown are representative photomicrographs of cells dual-stained with annexin-FITC/PI. Bar = 10 μm. **C.** Shown are representative photomicrographs of flow cytometric analysis; Statistical analysis of flow cytometric analysis. **D.** Western-blot of Bcl2 protein in A549 and H1299 cells after transfection; Statistical analysis of western-blot; qRT-PCR of Bcl2 mRNA in A549 and H1299 cells after transfection; Western-blot of cleaved-caspase-3 protein in A549 and H1299 cells after transfection; Statistical analysis of western-blot. Assays were performed in triplicate Means ± SEM are shown. Statistical analysis was conducted using student *t*-test.

Next, we examined the role of miR-329 on A549 and H1299 cells apoptosis. Firstly, we tested the caspase-3 and caspase-7 activity after treatment of A549 and H1299 cells with miR-329 mimic or miR-329 mimic NC, miR-329 inhibitor or miR-329 inhibitor NC, and results showed that miR-329 significantly increased the caspase-3 and caspase-7 activity in A549 and H1299 cell lysate, by approximately 13.5 and 9.0 folds increase (caspase-3 activity), 8.6 and 10.5 folds increase (caspase-7 activity), than that of bank A549 and H1299 cells (Figure 7A), respectively. However, loss of miR-329 by transfecting with miR-329 inhibitor remarkably reduced the caspase-3 and caspase-7 activity in A549 and H1299 cell lysate, by approximately 65% and 72% decrease (caspase-3 activity), 35% and 46% decrease (caspase-7 activity), than that of bank A549 and H1299 cells (Figure 7A), respectively. In addition, we also use dual-staining of annexin/PI to further evaluate the positive role of miR-329 in apoptosis of A549 cells. We found that miR-329 significantly promoted apoptosis in A549 and H1299 cells, while inhibition of miR-329 reversed the high level of apoptosis in comparison to miR-329 treated A549 and H1299 cells (Figure 7B), respectively. Further, our results of flow cytometric analysis demonstrated that forced expression of miR-329 resulted in a ~3-fold and ~2.4-fold increase in apoptotic cell death of A549 and H1299 cells (Figure 7C), respectively. However, the percentage of apoptotic cells induced by miR-329 was decreased to the basal level when the cells were treated with the specific miR-329 inhibitor (Figure 7C). Moreover, miR-329 also inhibited the expression level of anti-apoptotic protein Bcl2 (Figure 7D), and increased the protein expression of cleaved-caspase-3 (Figure 7D). These results demonstrated that miR-329 indeed promoted apoptosis in A549 and H1299 cells.

## DISCUSSION

Although oncogenic and tumor suppressive roles of several miRNAs have been characterized in several different types of tumors, the exact molecular mechanism by which miRNAs modulate the process of tumorigenesis is not yet fully elucidated. MiRNAs binding to the 3′-UTR of target mRNAs and in return leading to translational repression or degradation of mRNAs are well studied in recent years, and that may be an important approach to investigate their role on tumors. Down-regulation of miR-329 has been reported by miRNA profile studies on neuroblastoma [18-20], gastric cancer [21] and so on. These results reveal the powerfully inhibitory role of miR-329 on tumorigenesis. However, whether miR-329 plays a role on the development of lung cancer is still obscure.

In the present study, we firstly found that miR-329 was frequently down-regulated in human primary lung cancer tissues compared with matched normal lung tissues, and lower levels were found in NSCLC cell lines A549, H1299, SPC-A-1, SK-MES-1, 95-D and NCI-H520 in comparison to normal lung cell line HELF. Moreover, we also found that miR-329 inhibited the tumorigenic potential of lung cancer cells by down-regulating oncogenic targets. To our knowledge, this is the first report that reveals detail mechanism between loss of miR-329 and tumorigenic potential of lung cancer cells. This contention is based upon the following observations. First, miR-329 expression was significantly reduced in primary lung cancer. Second, miR-329 could directly regulate protein expression of c-Met by targeting the 3′-UTR of MET mRNA, and inhibit protein expression of cyclin D1 and gene expression of cyclin D1, cyclin D2, matrix metalloproteinase 7 (MMP-7) and MMP-9, while increase *p57* and *p21* gene expressions in lung cancer cell lines (A549 and H1299). Third, silence of *MET* gene repressed NSCLC cell (A549 and H1299) proliferation, migration, invasion and promoted cell apoptosis. Forth, expression of *MET* and miR-329 in 26 lung cancer patients indicated that there was an inverse correlation between miR-329 and *MET* levels. Fifth, over-expressing miR-329 ameliorated progression of NSCLC in an established experimental xenograft model. Finally, ectopic expression of miR-329 in the lung cancer cell lines (A549 and H1299) significantly reduced cell growth, migration, invasion and colony formation, and promoted cell apoptosis, whereas depletion of miR-329 remarkably promoted lung cancer cell (A549 and H1299) growth, migration, invasion and colony formation, and inhibited cell apoptosis. These findings suggest that miR-329 has an important role on inhibiting the development and progression of NSCLC.

Up to now, researches indicate miRNAs may function as oncogenes or tumor suppressors, just as protein-coding genes [24]. In fact, interactions between mRNA and miRNA are tightly regulated, and even a small change of these interactions may cause severe consequences to cell physiology [25]. Accordingly, alterations in the expression of target genes could lead to disease states. *MET* proto-oncogene is located on chromosome 7q21-31 and encodes the receptor tyrosine kinase MET. In several tumors, MET signaling pathway is aberrantly activated and represents one of the most important mechanisms of progression and invasiveness [26, 27]. Furthermore, aberrant MET signaling activation has been identified as a prognostic factor of poor outcome in different solid tumors and also in lung cancers [28-31]. In our present research, silence of *MET* expression inhibited lung cancer cell lines (A549 and H1299) growth, migration, invasion and promoted apoptosis. Further studies showed that exogenous miR-329 could down-regulate the expression of c-Met protein and mRNA in NSCLC cells. Moreover, the luciferase assay using a reporter containing the wild type miR-329 binding sequence at the 3′-UTR of *MET* indicated that the luciferase activity could be significantly reduced by over-expression of miR-329. Furthermore, the c-Met mRNA and protein were over-expressed in lung cancer tissues compared with normal tissues. Collectively, we discovered miR-329 targeted 3′-UTR of *MET*, and inhibited the expression levels of *c-Met* in lung cancer cell lines (A549 and H1299).

Then we examined the mechanism of miR-329 on lung cancer cell growth, and found that over-expression of miR-329 significantly inhibited A549 and H1299 cell proliferation, while loss of miR-329 promoted cell growth in A549 and H1299 cells. Further, miR-329 also suppressed tumor growth *in vivo*. In addition, we further showed that increase in miR-329 expression was associated with a decrease in oncogene cyclin D1, cyclin D2, and an increase in *p57* and *p21*. cyclin D1 and cyclin D2 are well-established oncogenes in many different cancers [32, 33]. It has been reported that over-expression of cyclin D1 promoted breast cancer cells (MDA-MB-236) [34] and lung cancer cells (A549) [35] proliferation. Our study showed that cyclin D1 and cyclin D2 were also up-regulated in the NSCLC cell lines (A549 and H1299). *p57* and *p21*, the cyclin-dependent kinase inhibitors, are considered to be a candidate of tumor suppressor gene that has been implicated in cancers [36, 37]. Our study revealed that the over-expression of miR-329 was associated with the up-regulation of p57 and p21 levels in NSCLC cells (A549 and H1299). Further, we also discovered miR-329 targeting 3′-UTR of MET mRNA, and inhibiting the expression of MET in lung cancer cells (A549 and H1299), which further contributing to the growth-delay efficacy of miR-329.

The present study also has shed light on the potential role of miR-329 on lung cancer metastasis. Metastasis is an integral part of tumor progression, and is a complex process attributed to loss of cellular adhesion, increased motility and invasiveness, entry and survival into the circulation, exit into new tissue, and eventual colonization of a distant site [38]. We showed that ectopic expression of miR-329 not only inhibited proliferation but also reduced cell migration and invasion motility of A549 and H1299 cells. MET is a receptor for hepatocyte growth factor and a tyrosine kinase (receptor-type tyrosine kinase), and supports the initial steps of invasion and metastasis of most human cancers, including lung cancer [39]. The down-regulation of MET could be a possible mechanism by which miR-329 regulates growth and metastatic potential of these cells. MMP-7 and MMP-9, which belongs to a family of zinc-dependent endopeptidases, are collectively capable of degrading essentially all of the components of the extracellular matrix (ECM), and are considered to be associated with invasion and migration of tumor cell [40, 41]. Our data supported the notion that the over-expression of miR-329 was associated with the down-regulation of MMP-7 and MMP-9 levels in lung cancer cells (A549 and H1299), which further confirmed the inhibitory role of miR-329 on invasion and migration of NSCLC cells.

We further investigated the role of miR-329 on NSCLC cell (A549 and H1299) apoptosis. Apoptosis is a stereotypical process of cell death intrinsic to all multicellular eukaryotic organisms and is critical for the elimination of unwanted, infected, or otherwise damaged cells [42]. The effectors of this process are caspases, proteolytic enzymes that drive cellular demolition within the cell. In our present study, we initially demonstrated that miR-329 promoted NSCLC cell (A549 and H1299) death, by targeting 3′-UTR of *MET*, inhibited the expression of anti-apoptotic protein Bcl2, and increased the expression of caspase-3 protein and increase of casepase-3/7 activities.

Our previous study reported miR-206 could also target 3′-UTR of MET and BCL2, and activate apoptosis, inhibit tumor cell proliferation, migration and colony formation in NSCLC cell lines (A549 and SK-MES-1) [46]. In fact, we have discovered several miRNAs (including miR-206, miR-139-5p, miR-329, miR-326 and miR-195-5p) were significantly down-regulated in NSCLC lung tissues in comparison to the adjacent normal lung tissues (Figure S1B). Among the five miRNAs, miR-329 and miR-206 could both target 3′-UTR of MET and exert tumor suppressor function in NSCLC. Thus, we thought there might be some connection between them, but there were no significance between the expression of miR-329 and miR-206 (Figure S2). Since miR-329 and miR-206 were not belonging to a same cluster, we thought their role on suppression the development of NSCLC might be individual.

In summary, we have shown that miR-329 is dramatically down-regulated in human lung cancer tissues compared with normal lung tissues. Moreover, up-regulation of miR-329 suppresses lung cancer cell migration, invasion and colony formation, and promotes lung cancer cell apoptosis, through targeting 3′-UTR of *MET*. In addition, miR-329 suppresses tumor cell growth *in vitro* and tumorigenicity *in vivo*. Collectively, our experimental data may provide a strategy for targeting the miR-329/*MET* interaction in a novel therapeutic application to treat lung cancer patients.

## MATERIALS AND METHODS

### Tissue collection

Lung cancer tissues and adjacent normal lung tissues were obtained from 26 patients who had undergone surgery at the People's Hospital of Wuhan University, between 2009 and 2011 and who were diagnosed with lung cancer based on histopathological evaluation (shown in Table 1). No local or systemic treatment had been conducted in these patients before the operation. All the tissue samples were collected, immediately snap frozen in liquid nitrogen, and stored at −80°C until RNA extraction. The study was approved by the Research Ethics Committee of Wuhan University (Wuhan, Hubei, PR China). Informed consent was obtained from all patients.

### Cell culture and transfection

The human NSCLC cell line, A549 and H1299, were grown in RPMI 1640 medium (Gibco, USA) containing 10% heat-inactivated (56, 30 min) fetal calf serum, 2 mmol/L glutamine, penicillin (100 U/mL) and streptomycin (100 U/mL), which was maintained in an incubator at 37°C with 5% CO_2_ in a humidified atmosphere. Hsa-miRNA-329 mimic and mimic negative control, hsa-miRNA-329 inhibitor and inhibitor negative control were purchased from RiboBio Co., Ltd. (Guangzhou, China). For convenience, hsa-miRNA-329 mimic and mimic negative control, hsa-miRNA-329 inhibitor and inhibitor negative control were simply referred to as miR-329 mimic and miR mimic NC, miR-329 inhibitor and miR inhibitor NC, respectively. Complete medium without antibiotics was used to culture the cells at least twenty four hours prior to transfection. The cells were washed with 1× PBS (pH7.4) and then transiently transfected with 50 nM miR-329 mimic or miR mimic NC, 100 nM miR-329 inhibitor or miR inhibitor NC, using Lipofectamine™ 2000 (Invitrogen, Carlsbad, CA, USA) according to the manufacturer's instructions.

### Western blot analysis

Forty eight hours after transfection, total protein was extracted from the A549 and H1299 cells using RIPA cell lysis reagent containing proteinase and phosphatase inhibitors (Solarbio) at 4°C for 30 min. Cell lysates were centrifuged at 12,000× g for 20 min at 4°C, and the protein concentrations of the supernatant were determined using the BCA protein assay reagent kit (Thermo) [44-47]. The supernatants containing total protein were then mixed with a corresponding volume of 5× SDS loading buffer and heated at 100°C for 10 min. Then, the supernatant lysates were run on 10% SDS-polyacrylamide gels (50 μg/lane), and proteins were transferred to poly (vinylidene fluoride) (PVDF) membranes (Hertfordshire, UK) by semidry electroblotting (1.5 mA/cm2). PVDF membranes were then incubated in blocking buffer [Tris-buffered saline (TBS) supplemented with 0.05% (vol/vol) Tween 20; TBST] containing 5% (wt/vol) skimmed milk powder for 120 min at room temperature followed by three 10 min washes in TBST. The PVDF membranes were then incubated with anti-c-Met (1:1000 dilutions, Affinity), anti-Bcl2 (1:1,000 dilutions, Affinity), anti-caspase3 (1:1,000 dilutions, Affinity), anti-cyclin D1 (1:1,000 dilutions, Affinity) and anti-GADPH (1:5,000 dilutions, Affinity) as internal normalizers in TBST containing 5% (wt/vol) skimmed milk powder (antibody buffer) overnight at 4°C on a three-dimensional rocking table. Then the membranes were washed three times for 10 min in TBST and then incubated with goat anti-rabbit IgG conjugated to horseradish peroxidase (1:12,000 dilutions) in antibody buffer for 120 min. Finally, membranes were washed three times for 10 min in TBST and exposed to ECL Advance reagent (GE Healthcare Biosciences, Buckinghamshire, UK) for 2 min as described in the manufacturer's protocol. Then membranes were exposed to Hyperfilm-ECL (GE Healthcare Bio-Sciences) for 2-5 min and visualized using a Fluor S Multimager and Quantity One 4.1 (Bio-Rad Laboratories, Hercules, CA ). The molecular weights of the bands were calculated by a comparison with prestained molecular weight markers (molecular weight range: 6,500 -250,000) that were run in parallel with the samples. Semiquantitative analysis of specific immunolabeled bands was performed using a Fluor S image analyzer and Quantity One 4.1.

### RNA isolation and quantitative reverse transcription poly-merase chain reaction (qRT-PCR)

Total RNA from the cultured cells was extracted using Trizol reagent (Invitrogen) according to the manufacturer's instructions. MiRNA levels were measured by qRT-PCR. For the qRT-PCR detection of mature miR-329 expression, we purchased the Bulge-Loop™ miRNA qRT-PCR Primer Set and the miRNA qRT-PCR Control Primer Set (both from RiboBio). RNA (2 μg) was converted into cDNA using the PrimeScript™ RT reagent kit with gDNA Eraser (Takara, Dalian, China) according to the manufacturer's instructions. qRT-PCR was performed using SYBR^®^ Premix Ex Taq™ II (Takara) in the ABI PRISM^®^ 7300 real-time PCR system (Applied Biosystems, Foster City, CA, USA). GADPH and U6 were used as endogenous controls. In addition, melting curves were used to evaluate non-specific amplification. The relative expression level was calculated using the 2^−ΔΔCt^ method. The primer sequences used in this study are presented in Table S1. The formula and its derivations were obtained from the ABI Prism 7300 sequence detection system user guide. Statistical analysis was performed on the fold change.

### Colony formation assay

Cells were transfected with miR-329 mimic or miR mimic NC, miR-329 inhibitor or miR inhibitor NC, as described above. Twenty four hours later, transfected cells were trypsinized, counted and replated at a density of 500 cells/6 cm dish. Ten days later, colonies resulting from the surviving cells were fixed with 3.7% methanol, stained with 0.1% crystal violet and counted. Colonies containing at least 50 cells were scored. Each assay was performed in triplicates.

### Luciferase reporter assays

The 3′-UTR of human MET were amplified from human genomic DNA and individually inserted into the pmiR-RB-REPORT^TM^ (Ribobio, Guangzhou, China) using the XhoI and NotI sites. Similarly, the fragment of MET 3′-UTR mutant was inserted into the pmiR-RB-REPORT^TM^ control vector at the same sites. For reporter assays, A549 and H1299 cells were co-transfected with wild-type (mutant) reporter plasmid and miR-Ribo^TM^ mimics (miR-Ribo^TM^ negative control) using Lipofectamine 2000 (Invitrogen). Firefly and Renilla luciferase activities were measured in cell lysates using the Dual-Luciferase Reporter Assay system. Luciferase activity was measured forty eight hours post-transfection using dual-glo luciferase reporter system according to the manufacturer's instructions (Promega, Madison, WI, USA). Firefly luciferase units were normalized against Renilla luciferase units to control for transfection efficiency.

### Transwell migration/invasion assay

A549 and H1299 cells were grown in RPMI 1640 medium containing 10% fetal bovine serum to ~60% confluence and transfected with 50 nM miR-329 mimic or a negative control, 100 nM miR-329 inhibitor or a negative control. After twenty four hours, the cells were harvested by trypsinization and washed once with Hanks' balanced salt solution (Invitrogen). To measure cell migration, 8-mm pore size culture inserts (Transwell; Costar, High Wycombe, UK) were placed into the wells of 24-well culture plates, separating the upper and the lower chambers. In the lower chamber, 500 μL of RPMI 1640 containing 10% FBS was added. Then, serum-free medium containing 5× 10^4^ cells were added to the upper chamber for migration assays, whereas 1×10^5^ cells were used for matrigel invasion assays. After twenty four hours of incubation at 37°C with 5% CO_2_, the number of cells that had migrated through the pores was quantified by counting 10 independent visual fields under the microscope (Olympus) using a ×20 magnifications, and cell morphology was observed by staining with 0.1% crystal violet. Filters were washed thoroughly with 1× PBS. Each experiment was performed at least three times.

### BrdU immunofluorescence assay

A549 and H1299 cells were seeded on sterile cover glasses placed in the 6-well plates. After transfection with miR-329 mimic, miR mimic NC, miR-329 inhibitor, miR inhibitor NC for forty eight hours, the BrdU (5-bromo-2-deoxyuridine; Sigma) stock solution at 10 mg/mL in saline was diluted 1000× in the culture medium and incubated for 60 min. After washing with PBS, cells were then fixed for 20 min in 4% paraformaldehyde (PFA) and permeabilized with 0.3% Triton X-100 for 10 min. After blocking with 10% goat serum in PBS for 1 h, cells were incubated with a primary rabbit antibody against BrdU (1:200, Abcam) over night at 4°C, and then incubated with the secondary antibody coupled to a fluorescent marker, Cy3, at room temperature for 2 h. After DAPI staining and PBS washing, the cover slips were mounted on to glass slides with anti-fade solution and visualized using a fluorescence microscope (Olympus 600 auto-biochemical analyzer, Tokyo, Japan) with Image-Pro Plus software for image analysis, and 10 microscopic fields were taken for calculating BrdU.

### CCK8 assay

Cell growth was measured using the cell proliferation reagent WST-8 (Roche Biochemicals, Mannheim, Germany). After plating cells in 96-well microtiter plates (Corning Costar, Corning, NY) at 1.0× 10^3^ /well, 10 μL of CCK8 was added to each well at the time of harvest, according to the manufacturer's instructions. Two hours after adding CCK8, cellular viability was determined by measuring the absorbance of the converted dye at 450 nm.

### Annexin V-FITC/PI analysis

A549 and H1299 cells were seeded on sterile cover glasses placed in the 6-well plates. After overnight growth, cells were transfected with miR-329 mimic, miR mimic NC, miR-329 inhibitor and miR inhibitor NC for forty eight hours. Then cells were washed with 1× PBS and fixed with 4% PFA for 10 min, and then incubated with Annexin V-FITC and Propidium (BestBio, Shanghai, China) following the manufacturer's instructions. Then they were finally observed under fluorescence microscopy (Olympus 600 auto-biochemical analyzer, Tokyo, Japan). Using Image-Pro Plus software (Media Cybernetics, Silver Spring, MD) to record images and analyze cell apoptosis.

### Flow cytometry

A549 and H1299 cells transfected with miR-329 mimic or negative control were trypsinized and resuspended in 1× binding buffer at 1× 10^6^ cells/mL. 100 μL of this cell suspension was incubated with 5 μL of FITC-Annexin V and 5 μL propridium iodide (PI) for 15 minutes in the dark. The reaction was terminated with the addition of 400 μL 1× binding buffer and analyzed with (FACSCalibur using the CellQuest software (Becton Dickinson). FITC-Annexin V-positive and PI-negative cells were considered as apoptotic and the experiments were carried out in triplicates.

### Wound healing assay *in vitro*

The A549 and H1299 cells were seeded in 6-well plates and incubated for twenty four hours, a linear wound was tehncreated by dragging a 100-μL pipette tip through the monolayer prior to transfection. Cellular debris was removed by gentle washes with culture medium, following which transfection was performed immediately, and the cells were allowed to migrate for a further forty eight hours. The healing process was dynamically photographed after the wound was introduced using a microscope (Olympus 600 auto-biochemical analyzer, Tokyo, Japan). Migration distance was measured from images (5 fields) taken at each indicated time point. The gap size was analyzed using Image-Pro Plus 6.0 software. The residual gap between the migrating cells from the opposing wound edge was expressed as a percentage of the initial gap size.

### Caspase-3/7 activity assay

The activity of caspase-3/7 was determined using the caspase-3/7 activity kit (Beyotime Institute of Biotechnology, Haimen, China). To evaluate the activity of caspase-3/7, cell lysates were prepared after their respective treatment with various designated treatments. Assays were performed on 96-well microtitre plates by incubating 10 μL protein of cell lysate per sample in 80 μL reaction buffer (1% NP-40, 20 mM Tris-HCl (pH 7.5), 137 mM Nad and 10% glycerol) containing 10 μL caspase-3/7 substrate (Ac-DEVD-pNA) (2 mM). Lysates were incubated at 37°C for 4 hours. Samples were measured with an ELISA reader at an absorbance of 405nm. The detail analysis procedure was described in the manufacturer's protocol.

### Transfection of siRNA

Three siRNA duplexes targeting human MET (GenBank accession no. NM_000245) were designed and synthesized by Guangzhou RiboBio Company (Guangzhou, China). For transfection, the cells were plated on an antibiotic-free growth medium at 60% confluence approximately twenty four hours before transfection. RNA oligonucleotides were transfected at a final concentration of 50 nM, using Lipofectamine 2000 (Invitrogen, USA) according to the manufacturer's protocol.

### Tumor formation in BALB/c nude mice

BALB/c athymic nude mice (male, 4-6-weeks old and 16-20 g) were purchased from Hubei Research Center of Laboratory Animal (Wuhan, China). All animal experiments were carried out in accordance with the Guide for the Care and Use of Laboratory Animals of Wuhan University. To establish lung cancer xenograft model, 5× 10^6^ A549 cells were suspended in 100 μL phosphate-buffered saline and inoculated subcutaneously into the flanks of nude mice. After 8 days, the transplanted nude mice were randomly divided into two groups (*n* = 6 each). miR-329 agomir (miR-329) or miR agomir NC (NC) (RiboBio Co., Ltd, Guangzhou, China) was directly injected into the implanted tumor at the dose of 1 nmol (in 20μL phosphate-buffered saline) per mouse every 4 days for seven times. The tumor size was monitored by measuring the length (L) and width (W) with calipers every 4 day, and the volumes were calculated using the formula: (L × W^2^)/2. Mice were killed by cervical dislocation after anaesthetized with 10% chloral hydrate in day 36, and the tumors were excised and snap-frozen for protein and RNA extraction.

### Statistical analysis

All experiments were repeated 3 times independently. The results are presented as the means ± standard error mean (SEM). Two independent sample *t*-test or One-Way Analysis of Variance (ANOVA) were performed using SPSS 19.0 software in order to detect significant differences in measured variables between or among groups. A value of *P* < 0.05 was considered to indicate a statistically significant difference.

## SUPPLEMENTARY MATERIAL FIGURES AND TABLE





## Abbreviations

miR: microRNA
miR-329: hsa-microRNA-329
NSCLC: non-small cell lung cancer
SCLC: small cell lung cancer
3′-UTR: 3′-untranslated region
nt: nucleotide
