# Supplementary material for: Hsa-miR-329 exerts tumor suppressor function through down-regulation of MET in non-small cell lung cancer
Source: Oncotarget. 2016 Feb 19;7(16):21510–26. doi: 10.18632/oncotarget.7517 (PMC5008302; doi:10.18632/oncotarget.7517)
Supplement: Supplementary file 1 [file oncotarget-07-21510-s001.pdf]

Hsa-miR-329 exerts tumor suppressor function through down-regulation of *MET* in non-small cell lung cancer

Supplementary Material

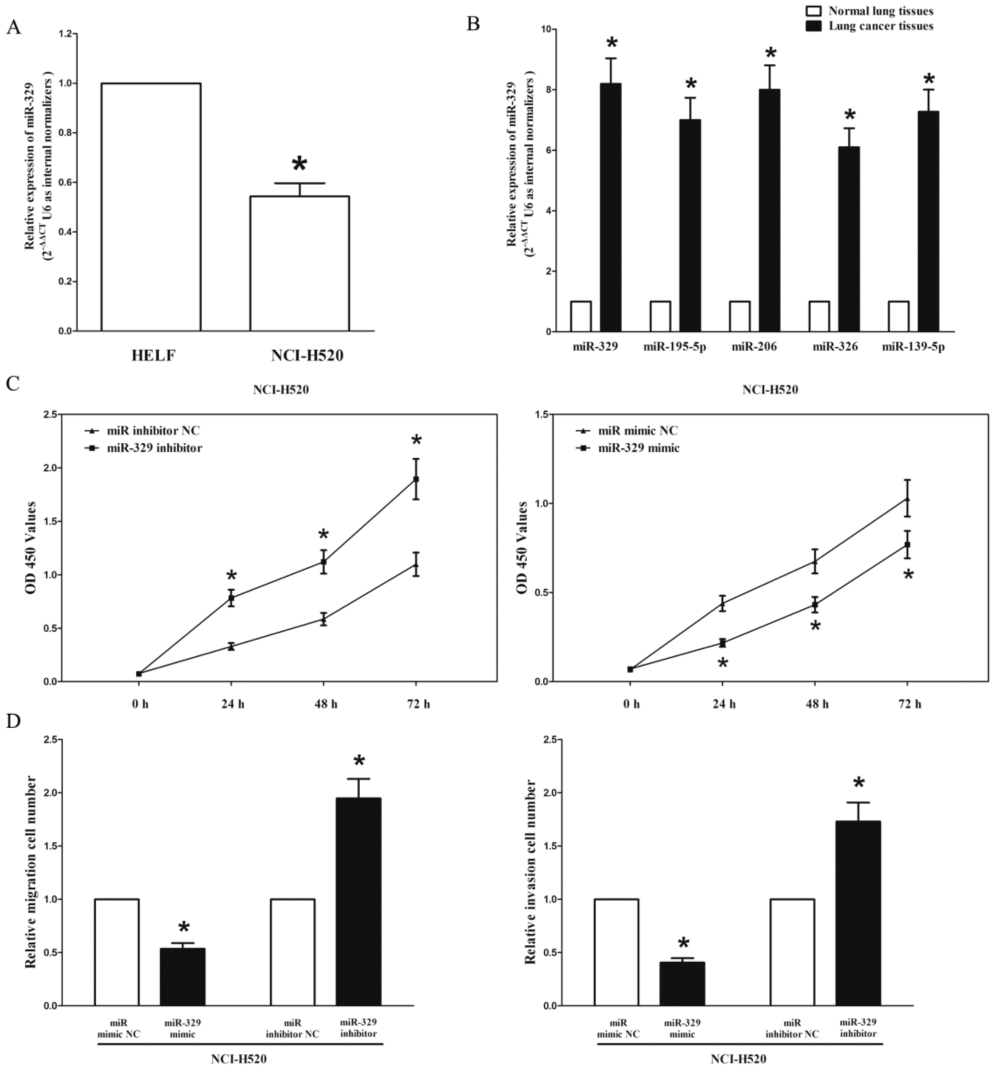

Fig. S1. The expression and role of miR-329 in NCI-H520 cells compared with HELF. **A**. The expression of miR-329 in HELF and NCI-H520 cells. **B**. The expression of miR-329, miR-206, miR-195-5p, miR-326 and miR-139-5p in primary lung cancer tissues and matched adjacent normal lung tissues. **C**. CCK8 assays of

NCI-H520 cells after transfected with miR-329 mimic, miR-329 mimic NC, miR-329 inhibitor, miR-329 inhibitor NC. **D**. NCI-H520 cells were loaded onto the top well of a transwell inserts for cell migration/invasion assay. After twenty four hours, cells that migrated to the bottom chamber containing serum-supplemented medium were stained with 0.1% crystal violet, visualized under a phase-contrast microscope, and photographed. Total number of cells in five fields was counted manually.

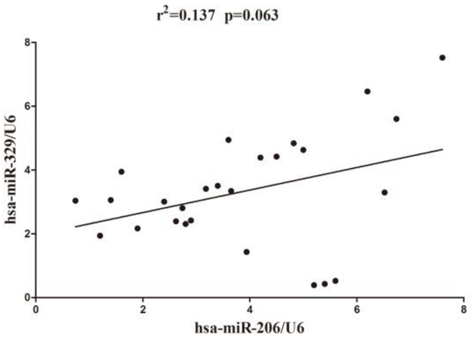

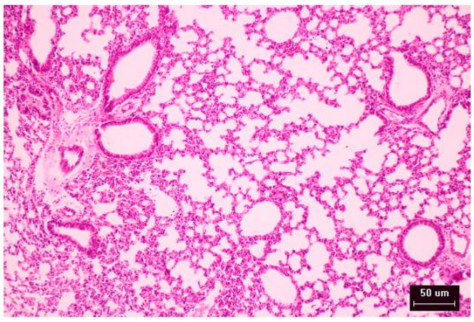

Adjacent normal lung tissues

Fig. S2. Scatter plots showing there is no significance between the expression of miR-329 and miR-206.
